# Health-related welfare prioritisation of canine disorders using electronic health records in primary care practice in the UK

**DOI:** 10.1186/s12917-019-1902-0

**Published:** 2019-05-22

**Authors:** Jennifer F. Summers, Dan G. O’Neill, David Church, Lisa Collins, David Sargan, David C. Brodbelt

**Affiliations:** 1 Royal Veterinary College, Hawkshead Lane, Hatfield, Hertfordshire AL9 7TA UK; 2 Faculty of Biological Sciences, University of Leeds, Leeds, LS2 9JT UK; 3 Department of Veterinary Medicine, University of Cambridge, Madingley Rd, Cambridge, CB3 0ES UK

**Keywords:** Canine, Welfare, Evidence-based, Breed-related, Prioritisation, Duration, Severity, Prevalence, VetCompass, EHR, EPR

## Abstract

**Background:**

Evidence-based comparison of the disorder-specific welfare burdens of major canine conditions could better inform targeting of stakeholder resources, to maximise improvement of health-related welfare in UK dogs. Population-level disease related welfare impact offers a quantitative, welfare-centred framework for objective disorder prioritisation, but practical applications have been limited to date due to sparse reliable evidence on disorder-specific prevalence, severity and duration across the canine disease spectrum. The VetCompass™ Programme collects de-identified electronic health record data from dogs attending primary-care clinics UK-wide, and is well placed to fill these information gaps.

**Results:**

The eight common, breed-related conditions assessed were anal sac disorder, conjunctivitis, dental disease, dermatitis, overweight/obese, lipoma, osteoarthritis and otitis externa. Annual period prevalence estimates (based on confirming 250 cases from total potential cases identified from denominator population of 455, 557 dogs) were highest for dental disorder (9.6%), overweight/obese (5.7%) and anal sac disorder (4.5%). Dental disorder (76% of study year), osteoarthritis (82%), and overweight/obese (70%) had highest annual duration scores. Osteoarthritis (scoring 13/21), otitis externa (11/21) and dermatitis demonstrated (10/21) highest overall severity scores. Dental disorder (2.47/3.00 summative score), osteoarthritis (2.24/3.00) and overweight/obese (1.67/3.00) had highest VetCompass Welfare Impact scores overall.

**Discussion:**

Of the eight common, breed-related disorders assessed, dental disorder, osteoarthritis and overweight/obese demonstrated particular welfare impact, based on combinations of high prevalence, duration and severity. Future work could extend this methodology to cover a wider range of disorders.

**Conclusions:**

Dental disorders, osteoarthritis and overweight/obese have emerged as priority areas for health-related welfare improvement in the UK dog population. This study demonstrated applicability of a standardised methodology to assess the relative health-related welfare impact across a range of canine disorders using VetCompass clinical data.

## Background

Breed-related disorders in dogs have been documented, discussed and investigated for well over a century [1–6] and concerns surrounding their negative effects on UK pedigree dog welfare are well established [7, 8]. Despite high profile media exposure and campaigns promoting public awareness of health issues, the popularity of purebred dogs as pets continues, with rising demand for certain ‘fashionable’ breeds regardless of well documented predispositions for particular health problems (e.g. pugs, French bulldogs) [9–13]. Recent independent inquiries called for further research and coordinated action to reduce the welfare burden of breed-related conditions on the pedigree dog population as a whole [5, 8, 14] and some progress has been made [15–17]. However, rationally identifying which of reportedly hundreds of breed-linked disorders should be prioritised to achieve greatest overall improvement in pedigree dog welfare from available resources remains a challenge [14, 18, 19]. This issue is even more complex when broadening the definition of ‘breed-associated’ disorders beyond those with evidence for direct genetic inheritance – it may be that certain conditions which could and do occur in any breed are far more likely in particular canine breeds or types, for epigenetic or owner-related environmental and socioeconomic reasons. In practical welfare terms, such conditions could also be considered ‘breed-associated’ to some degree, even if not strictly heritable or breed-exclusive.

Canine welfare is a dynamic discipline, with standards and definitions constantly evolving alongside ongoing animal welfare research [20–23]. There is apparent consensus between animal welfare scientists, canine welfare organisations and within UK legislation that health (incorporating, but not limited to, physical pain) is one of the most important elements contributing to the welfare of individual animals [24–27]. However, individual stakeholder opinions vary widely regarding the relative importance of specific areas of canine health-related welfare concern [28–32]. Evidence-based approaches to prioritising disorders for research and resource allocation are established in human medicine [33, 34] but there is currently no formal veterinary equivalent. A transparent, evidence-based strategy to identify, quantify and compare population-level welfare burdens of key canine conditions is required. Applied in a standardised way, outcomes of this objective comparison tool could inform strategic targeting of available stakeholder resources, optimising overall canine health-related welfare improvement [35, 36].

Emerging concepts/tools in animal welfare epidemiology now offer an ethically and scientifically sound conceptual framework for disorder comparison according to relative health-related welfare impact at the population level [35, 36]. Historically, canine welfare assessment scales proposed were disorder-specific and applicable at individual dog, rather than population, level [37–41]. Efforts to address this within the animal welfare science community are progressing, with development of metrics designed to quantify and compare detrimental welfare effects across a range of canine disorders, taking into account various aspects of welfare impairment. Examples include the Generic Illness Severity Index for Dogs (GISID; a measure of relative severity for canine inherited diseases) [14], Breed-Disorder Welfare Impact Score (BDWIS; a measure of welfare impact on dogs with particular inherited diseases) [36] and more recently, Welfare-Adjusted Life Years (WALYs) [42]. Welfare impact assessment takes into account measures of disorder prevalence, severity and duration to report and compare the welfare importance of individual disorders for a given population, providing a quantitative, welfare-centric basis for objective disorder prioritisation [35]. Practical applications of this approach are established in production and laboratory animal welfare scoring systems [43, 44]. To date, suggested application of the concept to pet dog populations has been limited by a dearth of reliable, disorder-specific evidence on prevalence, duration and severity of effect of specific conditions in different breeds [14, 18, 35, 36]. Furthermore, large-scale datasets relevant to pet dog health have been unavailable due to commercial sensitivity, or else impossible to interrogate using existing validated tools. The VetCompass animal health surveillance programme holds clinical electronic health record (EHR) data from over 5 million dogs attending over 1000 primary-care clinics UK-wide, and is well placed to fill information gaps relevant to disorder-specific welfare impact using clinical and demographic information that is both large-scale and geographically representative [45].

### Aims and objectives

This project aimed to provide an evidence-led basis for prioritising action on certain canine breed-related disorders over others, according to comparative health-related welfare impact. Broadly, the objectives were to:

1. Develop a data-driven strategy for disorder prioritisation, using novel welfare metrics to evaluate the data available in routinely collected canine EHR.
2. Apply this strategy to a defined set of relevant disorders, providing transparent evidence to support particular disorders as priorities for reform.

## Results

The eight disorders selected for assessment of relative health-related welfare impact were anal sac disorder, conjunctivitis, dental disorder, dermatitis, lipoma, osteoarthritis, otitis externa and overweight/obese. Disorder groupings were based on previously published VetCompass analyses estimating prevalence of disorders recorded in dogs attending primary-care veterinary practices in England [46]. The denominator study population comprised 455,557 dogs under the care of 304 VetCompass participating clinics distributed throughout the UK.

### Health related welfare metrics

#### Annual period prevalence

All-breed annual period prevalence estimates with 95% confidence intervals for the eight disorders assessed are presented in Table 1. Dental disorder (9.6%), overweight/obese (5.7%) and anal sac disorder (4.5%) had highest estimated prevalences for this annual period.

**Table 1:** All-breed annual period prevalence estimates for eight common disorders of UK dogs with evidence for breed-related status

Disorder assessed	All-breed annual period prevalence estimate (%) [95% CI]	Breeds with numerical over-representation in disorder case group compared with background VetCompass population				
		Breed/type	N (of 250 study cases)	Breed-specific annual period prevalence estimate (%)	95% CI (%)	Prevalence ratio (Breed-specific prevalence:All-breed prevalence)
Dental disorder	9.6 [8.5–10.8]	Greyhound	9	50.0	(26.8–73.0)	5.2
		CKCS	14	24.2	(13.1–35.2)	2.5
		Border terrier	6	19.3	(5.4–33.1)	2.0
		Bichon	7	18.6	(6.2–31.0)	1.9
		Chihuahua	11	16.4	(7.5–25.1)	1.7
		Cocker spaniel	14	14.9	(7.7–22.1)	1.6
		Springer spaniel	9	14.1	(5.6–22.6)	1.5
		Yorkshire terrier	12	13.7	(6.5–20.8)	1.4
		JRT	19	12.0	(7.0–17.1)	1.3
		WHWT	8	11.7	(4.1–19.3)	1.2
Overweight / obesity	5.7 [5.0–6.4]	Beagle	6	17.8	(4.9–30.7)	3.1
		CKCS	11	11.3	(5.0–17.6)	2.0
		Labrador	34	10.6	(7.2–14.0)	1.9
		Pug	5	9.7	(1.6–17.7)	1.7
		Golden retriever	5	9.6	(1.6–17.5)	1.7
		Border terrier	5	9.5	(1.6–17.5)	1.7
		Bichon	6	9.4	(2.3–16.6)	1.7
		Cocker spaniel	14	8.9	(4.4–13.3)	1.6
		JRT	22	8.3	(5.0–11.6)	1.5
		WHWT	9	7.8	(2.9–12.7)	1.4
		Springer spaniel	8	7.4	(2.5–12.4)	1.3
Anal sac disorder	4.5 [4.0–5.0]	CKCS	23	18.7	(11.8–25.6)	4.1
		Bulldog	6	14.6	(3.8–25.4)	3.2
		Shih-tzu	20	11.0	(6.4–15.5)	2.4
		Bichon	6	7.5	(1.7–13.2)	1.7
		Cocker spaniel	13	6.8	(3.2–10.3)	1.5
		Lhasa Apso	5	6.0	(0.9–11.1)	1.3
		WHWT	8	5.5	(1.8–9.2)	1.2
		Springer spaniel	7	5.2	(1.4–8.9)	1.1
Otitis externa	4.0 [3.5–4.5]	KCS	5	16.6	(3.3–29.9)	4.2
		WHWT	15	8.6	(4.5–12.8)	2.2
		Pug	5	8.1	(1.3–14.9)	2.1
		Cocker Spaniel	17	6.6	(3.6–9.7)	1.7
		Labrador	35	6.0	(4.1–8.0)	1.5
		Springer Spaniel	8	6.0	(2.0–10.0)	1.5
		GSD	11	5.6	(2.4–8.8)	1.4
		CKCS	7	5.0	(1.4–8.5)	1.3
Dermatitis	3.6 [3.1–4.0]	Bulldog	6	11.5	(2.9–20.2)	3.2
		Border terrier	6	7.2	(1.6–12.7)	2.0
		Bichon	7	6.9	(2.0 - 11.8)	1.9
		GSD	13	6.7	(3.2–10.3)	1.9
		Boxer	6	6.2	(1.4–11.0)	1.7
		Pug	5	6.0	(0.9–11.2)	1.6
		Labrador	26	5.1	(3.2–7.0)	1.4
		WHWT	9	4.9	(1.8–8.0)	1.4
		Cocker Spaniel	12	4.7	(2.1–7.4)	1.3
		Springer Spaniel	8	4.6	(1.5–7.8)	1.3
		Shih-tzu	10	4.3	(1.7–6.9)	1.2
Osteoarthritis	2.3 [2.1–2.6]	Golden retriever	10	7.9	(3.2–12.5)	3.4
		Labrador	52	6.7	(4.9–8.4)	2.8
		Rottweiler	8	6.4	(2.1–10.7)	2.7
		GSD	17	5.8	(3.1–8.5)	2.5
		Border collie	10	3.5	(1.4–5.7)	1.5
		CKCS	8	3.4	(1.1–5.7)	1.4
Conjunctivitis	2.3 [2.0–2.6]	CKCS	17	7.1	(3.8–10.3)	3.1
		Labrador	28	3.5	(2.3–4.8)	1.5
		WHWT	10	3.5	(1.4–5.7)	1.5
		Cocker spaniel	11	2.9	(1.2–4.6)	1.3
Lipoma	1.2 [1.1–1.3]	Labrador	47	3.1	(2.2–4.0)	2.6
		Springer spaniel	14	2.8	(1.3–4.2)	2.3
		Golden retriever	6	2.4	(0.5–4.3)	2.0
		Border collie	12	2.2	(1.0–3.4)	1.8

Abbreviations: *95% CI* 95% confidence interval, *WHWT* West Highland White Terrier, *KCS* King Charles Spaniel, *CKCS* Cavalier King Charles Spaniel, *GSD* German Shepherd dog, *JRT* Jack Russell terrier

Within disorders breed-specific annual period prevalence estimates are listed for breeds represented by 5 or more individuals in the study group and where estimated breed-specific prevalence: all-breed prevalence ratios indicated breed over-representation compared with the VetCompass denominator population

#### Duration

Otitis externa, anal sac disorder, conjunctivitis and dermatitis all showed a median of one episode per case over the year, with maximum annual episode counts varying from three (conjunctivitis) to seven (anal sac disorder) (Table 2). Neither median annual number of episodes or median episode duration were calculated for osteoarthritis on the assumption that, once present, this disorder was continuously present until death (non-episodic) rather than intermittent/episodic. The proportion of cases diagnosed with more than one episode during the study year was thus reported as ‘Not applicable’ for osteoarthritis. It was not possible to reliably calculate median annual number of episodes or median episode duration for dental disorder, overweight/obese or lipoma groups as so few cases had sufficiently clear data available on both episode diagnosis and resolution dates. Proportion of cases diagnosed with more than one episode was reported as zero for these three disorders, as, despite the biological plausibility of multiple episodes (e.g. total removal and subsequent recurrence of lipoma, fluctuation between obesity and healthy bodyweight) there was insufficiently clear data available to confirm multiple episodes in any case (Table 2).

**Table 2:** Metrics reflecting comparative average duration for eight common disorders of UK dogs with evidence for breed-related status

	Median annual duration				Episodes per case during study year					Episode duration					Median age at first diagnosis/disorder-related visit during study period			
	Median % of year affected	Interquartile range (%)	Range (%)	*n (dogs)*	Median episodes	Interquartile range	Maximum	% cases with > 1 episode/year	*n (dogs)*	Median days/episode	Interquartile range (days)	Range (years)	% (n) cases affected all year	*n episodes* *[dogs]*	Age (years)	Interquartile range (years)	Range (years)	*n (dogs)*
Osteoarthritis	81.9	48.0–100	1.4–100	*250*	*N/A (non-episodic)*			*N/A*	*250*	*N/A (non-episodic)*			38.4 (96)	*N/A*	9.9	7.3–12.1	1.0–16.0	*250*
Dental disease	76.3	32.7–100	0.3–100	*244*	*N/A (Data insufficient)*			*0*	*244*	*N/A (Data insufficient)*			39.9 (97)	*N/A*	6.0	3.3–9.1	0.2–17.4	*248*
Overweight/obesity	69.6	31.4–100	0.3–100	*247*	*N/A (Data insufficient)*			*0*	*247*	*N/A (Data insufficient)*			30.0 (74)	*N/A*	5.2	3.0–7.8	0.2–14.5	*249*
Lipoma	54.5	23.2–99.6	0.3–100	*243*	*N/A (Data insufficient)*			*0*	*243*	*N/A (Data insufficient)*			25.1 (61)	*N/A*	9.3	7.5–11.3	1.3–17.9	*245*
Otitis Externa	3.8	*N/A*	0.8–65.8	*76*	1	1–1	4	12.4	*250*	14	8–23	3–60	0	*79 [76]*	5.1	2.2–8.8	0.2–15.4	*250*
Dermatitis	3.8	*N/A*	0.8–100	*62*	1	1–1	5	9.6	*250*	14	10–39	3–175	0	*62 [59]*	4.2	1.9–7.4	0.2–15.0	*242*
Anal Sac disorder	3.8	*N/A*	0.5–100	*23*	1	1–2	7	25.6	*250*	14	7–22	2–82	0	*23 [20]*	4.0	2.0–7.5	0.2–16.4	*250*
Conjunctivitis	2.2	*N/A*	0.5–100	*46*	1	1–1	3	7.2	*249*	8	5–14	2–365	1.2 (3)	*46 [46]*	4.0	1.3–8.0	0.1–15.6	*248*

Metric values are at disorder-level, based on analysis of group data obtained via structured review of electronic health records from 250 affected dogs per disorder during 2013. Disorders presented in descending order of median annual duration

For osteoarthritis, lipoma, dental disorder, overweight/obese and conjunctivitis the proportion of cases affected for the entire study period ranged from 39.9% (dental disorder) to 1.2% (conjunctivitis, based on 3 cases diagnosed with keratoconjunctivitis sicca before 01.01.2013) (Table 2). Across the eight disorders, the median annual duration (median % of year affected) ranged from 81.9% in the osteoarthritis group to 2.2% for conjunctivitis cases (Table 2).

Median ages at earliest disorder-related visit during the year for the eight conditions were 4.0 years for anal sac disorder and conjunctivitis, 4.2 for dermatitis, 5.1 for otitis externa, 5.2 for overweight/obese, 6.0 for dental disease, 9.3 for lipoma and 9.9 years for osteoarthritis (Table 2). Within-disorder interquartile range (IQR) for this measure was widest for conjunctivitis (6.7 years) and narrowest for lipoma (3.8 years). Data for this measure were complete (i.e. available for all 250 study dogs) in osteoarthritis, otitis externa and anal sac disorder case groups, with lowest data availability in the dermatitis group (245 dogs contributing data).

#### Severity

Table 3 presents individual metric and overall severity scores for each disorder. Overall severity scores (maximum possible score of 21) were highest for osteoarthritis (13/21 score), followed by otitis externa (11/21), dermatitis (10/21), conjunctivitis (9/21), lipoma (8/21), anal sac disorder and dental disorder (7/21) and overweight/obese (3/21).

**Table 3:** Disorder-level severity scoring for eight common disorders of UK dogs with evidence for breed-related status, including individual severity sub-scores and summative overall severity score

Disorder assessed	Categorical disorder-level severity metric sub-scores based on case group data over study year (categories represented by sub-scores 0–3 [see Table 5])							Overall disorder-level severity score
	*Severity metric 1:* Disorder as reason presented for veterinary care during study year	*Severity metric 2:* Frequency of disorder-related primary care clinic visits during study year	*Severity metric 3:* Chronicity of disorder-related analgesia (including AI) therapy during study year	*Severity metric 4:* Range of other (non-analgesic/AI) therapeutic groups used for disorder treatment/management in study year	*Severity metric 5:* Surgical depth of any disorder-related procedures performed under GA/sedation during study year	*Severity metric 6:* Disorder-related overnight primary-care hospitalisations during study year	*Severity metric 7:* Disorder-related referral during study year	
Osteoarthritis	3	2	3	1	1	0	3	13
Otitis Externa	3	1	2	3	1	0	1	11
Dermatitis	3	2	2	2	1	0	0	10
Conjunctivitis	3	1	1	2	1	0	1	9
Lipoma	2	1	1	1	1	1	1	8
Anal sac disorder	3	1	1	1	1	0	0	7
Dental disease	1	1	1	1	1	1	1	7
Overweight/obese	1	1	0	1	0	0	0	3

Abbreviations: *AI* Anti-inflammatory, *GA* General anaesthesia

Sub-score allocation is at the disorder-level, based on application of the severity metric scoring criteria (see Table 5) to group data obtained via structured review of electronic health records from 250 affected dogs per disorder during 2013

#### VetCompass welfare impact score

As the highest scoring disorders assessed, dental disorder (prevalence 9.62%) and osteoarthritis (duration 81.9%; overall severity score 13/21) provided reference values for calculation of relative prevalence, duration and severity index scores across conditions. Dental disorder ranked highest for the VetCompass Welfare Impact score (VWI score 2.47) followed by osteoarthritis (2.24), overweight/obese (1.67), lipoma (1.41), otitis externa (1.30), dermatitis (1.19), anal sac disorder (1.05) and conjunctivitis (0.96) (Table 4).

**Table 4:** Comparison of key health-related welfare impact metrics for eight common disorders of UK dogs with evidence for breed-related status

	PREVALENCE	DURATION	SEVERITY (individual metric sub-scores: 0–3 [none – most severe]; Overall score: maximum 21)								HEALTH RELATED WELFARE IMPACT
	All-breed annual UK period prevalence estimate (%)	Median annual disorder duration (% of year affected)	Severity metric 1: Disorder as reason presented for primary-care veterinary attention	Severity metric 2: Frequency of disorder-related primary-care veterinary clinic visits	Severity metric 3: Disorder-related analgesia/anti-inflammatory therapy	Severity metric 4: Disorder-related non-analgesic/anti-inflammatory intervention groups	Severity metric 5: Disorder-related procedures under general anaesthesia/sedation	Severity metric 6: Disorder-related overnight hospitalisations	Severity metric 7: Disorder-related referral	Overall Disorder Severity score	VetCompass Welfare Impact score
Dental disease	9.6	76.4	1	1	1	1	1	1	1	7	2.47
Osteoarthritis	2.3	81.9	3	2	3	1	1	0	3	13	2.24
Overweight/obesity	5.7	69.6	*1*	*1*	*0*	*1*	*0*	*0*	*0*	3	1.67
Lipoma	1.2	54.5	*2*	*1*	*1*	*1*	*1*	*1*	*1*	8	1.41
Otitis Externa	4.0	3.8	*3*	*1*	*2*	*3*	*1*	*0*	*1*	11	1.30
Dermatitis	3.6	3.8	*3*	*2*	*2*	*2*	*1*	*0*	*0*	10	1.19
Anal sac disorder	4.5	3.8	*3*	*1*	*1*	*1*	*1*	*0*	*0*	7	1.05
Conjunctivitis	2.3	2.2	*3*	*1*	*1*	*2*	*1*	*0*	*1*	9	0.96

Disorders presented in descending order of VetCompass Welfare Impact score

#### Numerical over-representation of individual breeds within disorder-specific study case groups

In each disorder a number of breeds showed numerical over-representation as evidenced by breed-specific:all-breed prevalence ratios greater than one, with a number of breeds additionally demonstrating no overlap of 95% confidence intervals for breed-specific and all-breed prevalence estimates in each disorder other than for dermatitis (Table 1). Prevalence ratios greater than two were seen for the following breeds in the stated disorder study groups: Greyhound, CKCS and Border terrier in dental disorder (ratios 5.2, 2.5, 2.0 respectively), Beagle and CKCS in overweight/obese (3.1, 2.0), CKCS, Bulldog and Shih-tzu in anal sac disorder (4.1, 3.2, 2.4), KCS, WHWT and Pug in otitis externa (4.2; 2.2; 2.1), Bulldog and Border terrier in dermatitis (3.2, 2.0), Golden retriever, Labrador, Rottweiler and GSD in osteoarthritis (3.9, 2.8, 2.7, 2.5), CKCS in conjunctivitis (3.1), Labrador, Springer spaniel and Golden retriever in lipoma (2.6, 2.3, 2.0).

## Discussion

Improving the health-related welfare of dogs requires identification and prioritisation of important health issues for targeted reform. Breed-related disorders are established as a significant issue affecting UK dog welfare, but identifying specific disorders on which to focus available resources for maximum canine welfare improvement is challenging [8, 14, 18]. This paper describes the development and application of a VetCompass data-driven strategy for evidence-based canine disorder prioritisation based on comparing relative health-related welfare impact. Novel welfare metrics, developed to assess annual frequency, severity and duration across disorders, were generated for eight major disorders with evidence of breed associations, using EHR data from randomly selected cases identified from VetCompass. Comparable, group-level disorder prevalence, severity and duration scores were combined into novel VetCompass Welfare Impact scores, reflecting relative disorder-specific, population-level welfare impact and highlighting areas of particular welfare concern for potential targeted reform [35, 36]. The described methods for data-driven, health-related welfare metric generation and comparison are applicable across the disorder spectrum. This offers opportunities for future studies to both expand the reported eight disorder comparison, or to objectively assess, compare and suggest priorities within other disorder groups.

### Disorders assessed

Breed-related disorders given highest media exposure or cited as particularly concerning by expert panels on pedigree dog health are often those perceived as especially severe for affected individuals within certain high-risk breed groups [8, 32]. Some of these issues have extreme animal-level welfare implications, being present from birth to death, clinically unresolvable and difficult/impossible to manage through owner education and/or changes to practice. While important, such disorders may however be relatively uncommon within dog populations as a whole and priority should arguably be given to disorders with the greatest welfare burden across canine populations.

This study used prevalence estimates generated from VetCompass data [46] to identify the most common health issues of welfare impact with any breed associations, shifting the focus of prioritisation toward issues with greatest health-related welfare burden across UK dogs [35, 36]. All eight disorders assessed had sufficient existing evidence to score at least two on the SEHB scale [14, 18, 19], supporting likely breed-related as a primary disorder or secondary to disorders with breed-associations (e.g. dermatitis secondary to atopy [47]). Despite lower media profiles and historically poorer research focus [48] several of these (e.g. bodyweight/obesity and dental disorders) have been highlighted by recent UK surveys as health and welfare concerns of veterinary surgeons and other UK canine welfare stakeholders [29, 49–51].

### Prevalence

Of the disorders studied, dental disorders (9.6%), overweight/obese (5.7%) and anal sac disorders (4.5%) had the highest prevalence and showed results that were broadly in line with previous reports [46, 52]. The prevalence values reported in the current study were generally lower than previous estimates that were often based on referral populations that may have been selectively biased towards sicker populations. Nonetheless, these estimates may also reflect under-reporting of certain conditions such as overweight/obese or osteoarthritis by primary-care veterinarians as well as differences in case definition between this and other studies [42, 46]. As applied here, prevalence represented a reproducible and comparable metric providing an annual ‘snapshot’ of relative disorder frequency across the study population as a whole and within breed groups. Whilst the absolute frequencies may be underestimates, the relative order of conditions was likely to be a reliable indicator of their relative disease burden. Building on previous disorder-specific work on data from VetCompass, it provided a systematic basis for disorder groupings which could realistically be investigated using these large-scale, primary care EHR datasets [46].

### Duration

Duration of individual health problems is increasingly emphasised by animal welfare scientists as a key contributor to the overall welfare impact, as recognised in human medicine [23]. Disorders affecting dogs for extended periods pose greater risk of cumulative or lifelong pain and other welfare issues, even where daily levels of discomfort/pain, debilitation or distress appear relatively low. A recent report by Teng et al. proposed and applied the Welfare-Adjusted Life Year (WALY), adapted from the human Disability-Adjusted Life Year (DALY) concept, as a measure of cause-specific welfare impact on individual dogs [53]. This complex metric puts strong emphasis on disorder duration as a component of overall welfare compromise, taking into account both time lived with impaired welfare due to a given cause and years of life lost after premature death from that cause (with weighting by perceived cause-specific level of welfare compromise). Application to 10 common disorders demonstrated that those with the greatest and least adverse impact on dogs, according to magnitude of WALY, were atopic dermatitis (WALY 9.73, largely attributable to time lived impaired) and thoracolumbar intervertebral disc disease (WALY 2.83, mainly comprised of years of life lost). It is also worth noting that the course of many chronic, progressive disorders involves episodic “steps”, where issues increase to a threshold which precipitates revisits and additional intervention. Recognising and promoting better management of chronic conditions at these times provides opportunities to significantly reduce welfare burdens associated with chronic disease that may have been historically underestimated [54].

Based on the median age at earliest disorder-associated record, dermatitis, anal sac disorders and conjunctivitis appeared to affect a relatively younger demographic than osteoarthritis and lipoma. Although late-onset (geriatric) health issues are often cited as of particular concern [55, 56], it could also be argued that chronic or progressive disorders routinely affecting dogs from an early age constitute a greater priority concern due to their potentially lifelong welfare effects.

Osteoarthritis, dental disorder, lipoma and overweight/obese had median annual duration estimates that exceeded 50, 60, 70 and 80% respectively with over a quarter of cases affected for the entire annual period. In contrast, otitis externa, dermatitis, anal sac disorder, conjunctivitis had much lower median durations (< 4%). In the latter group of conditions, the number of episodes per case could generally be more reliably determined than individual episode durations. Ambiguous or absent revisit data introduced degrees of uncertainty and subjective interpretation when time delimiting distinct episodes, with median duration often based on only small numbers of episodes. Even where feasible episode ‘start’ and ‘end’ dates were available, these likely underestimated true duration as disorders were likely clinically present before the diagnostic visit. In addition, the median duration metric itself potentially underestimated the longer term, cumulative importance of seasonally recurring episodic disorders. Annual recurrence patterns seen in seasonally triggered allergic skin disorders can manifest as a single, annual ‘flare up’ of dermatitis, otitis externa, conjunctivitis and/or anal sac irritation [47, 57]. This single, seasonal episode per study year generates a low median duration which may not properly reflect cumulative lifetime welfare burden for affected individuals. In the context of the present retrospective study design and data, it was not possible to completely address these limitations. However, median duration could theoretically be assessed with greater accuracy within prospective clinical studies, gathering reliable episode duration through planned progress assessments and welfare impact metrics could be generated using longer defined study periods to explore potential limitations for seasonally linked or infrequently recurrent disorders.

Objective and quantitative methods are required to compare duration across the spectrum of canine disease. The current study estimated the annual duration for each disorder but given that this was across a sample of 250 cases, it also reflected the varying age structures of these cases. Annual disorder duration could also be useful to estimate the lifetime duration for each disorder although this remains challenging until large-scale birth-to-death health data on dogs become available. In the meantime, the novel median duration metric developed in the current study offers a reproducible, comparable alternative to true lifetime duration measures (or WALYs), by restricting retrospective review of EHR data to a single study year and using information typically recorded in these datasets across a wide spectrum of disorders.

### Severity

Overall severity scores were highest for osteoarthritis (score 13), otitis externa [11] and dermatitis [10]. Over 50% of osteoarthritis cases presented primarily for the condition at least once, over 50% received over 14 consecutive days of analgesic/anti-inflammatory therapy and over 3% were referred for osteoarthritis management in the study year. This suggests that osteoarthritis is recognised and perceived by both veterinary surgeons and owners as important enough for significant clinical care, often involving long term maintenance prescription only analgesia, multiple additional medical interventions, frequent clinic visits and relatively high levels of referral uptake. Severity scores were marginally lower but showed similar patterns in otitis externa and dermatitis. However, the particularly high sub-score assigned to otitis externa for non-analgesic/non-anti-inflammatory therapy prescribed was influenced by the formulation of many aural medication products, which typically contain anti-septic, anti-microbial, anti-fungal and physical cleansing elements in a single preparation.

Overweight/obese and dental disorders had comparatively low overall severity scores (3 and 7), which appears contradictory to established veterinary consensus on the critical importance of these issues to health-related canine welfare [29, 49–51]. Diagnosis was usually incidental, with few associated veterinary visits and minimal interventions received, suggesting that owners may fail to recognise these problems or are not sufficiently motivated or concerned to seek veterinary attention or take up recommended interventions. A 2011 online survey reported that only 52.3% of owners would present a pet primarily for halitosis, supporting low owner recognition or appreciation of the serious clinical consequences associated with dental disorders [55]. Alternatively, owners may be unaware of treatment possibilities, unconvinced of the need for dental care/weight loss in their pets or confused by conflicting advice from different areas of the pet health care sector. Owner choices may be based on external and non-veterinary advice, or financial restrictions or priorities, particularly if treatment or care is excluded by pet insurance policies. Some may be unwilling or unable to invest the time or find recommended day-to-day interventions like tooth brushing and dietary restriction difficult to implement [58–60].

Owner uptake (not just veterinary recommendation) of interventions, re-examination or referral were key when assigning disorder-specific sub-scores for individual severity metrics. This helps reflect owner perceptions of disorder importance but could mask veterinary-perceived severity in disorders where owners frequently declined recommended interventions or failed to return for follow-up care, perhaps due to lack of concern or limited resources. As applied here, the severity scoring system did not consider the welfare consequences of common secondary conditions or exacerbation of co-morbidities when assigning severity scores for assessed disorders. For example, no overweight/obese cases received directly therapeutic referral, analgesia/anti-inflammatories or procedures under GA/sedation, but potentially important detrimental consequences of sequelae/co-morbidities such as osteoarthritis [61–64] also remained unaccounted for in cases. Specific co-morbidity scenarios could theoretically be evaluated and compared in future studies, providing transparent case definitions were clearly defined. Overall, comparison of sub-scores for the seven severity metrics gave useful insights into owner-perceived importance of different disorders, by reflecting differential levels of drive to present affected dogs, take up recommended interventions and attend for ongoing or specialist care.

### Overall welfare impact and suggested priority areas

Dental disorder, osteoarthritis, overweight/obese and lipoma ranked highest among the 8 studied disorders based on VetCompass Welfare Impact scores. Top-ranking duration and severity scores, interpreted alongside individual severity metric sub-scores characterised osteoarthritis as a relatively frequent primary reason for veterinary presentation, with multiple related visits per year, frequent association with chronic pain management and a notable level of associated referral. This evidence supports osteoarthritis as a priority disorder for targeted reform [42].

Recent surveys by the British Veterinary Association (BVA) and Peoples Dispensary for Sick Animals (PDSA) reported that UK veterinary surgeons consider obesity/excessive bodyweight [29, 49, 51] and dental disorders [50] to be the most important health related welfare issues currently affecting UK dogs. Both scored highly for prevalence and duration, but severity sub-scores appeared to indicate mismatches in veterinarian-owner perceptions of their individual welfare importance. This may also highlight dental disorders and overweight/obese as areas where increasing owner awareness of clinical signs, primary consequences and secondary risks of associated sequelae could improve health-related welfare in a significant proportion of UK dogs [65, 66].

### Numerical over-representation of individual breeds within disorder case groups

A number of breeds were numerically over-represented in every disorder group. Assessing breed over-representation within a disorder by comparing breed-specific annual period prevalences with overall period prevalence was intended to avoid highlighting spurious potential disorder-associations in generally popular breeds (i.e. some breeds may present more with dental disorders simply as a function of their popularity within the population, but are not numerically over-represented within the randomly selected case group studied). The authors acknowledge that these comparisons were only undertaken for the most commonly represented breeds in each disorder (represented by > 5 individuals), thus less likely to flag-up potential disorder associations in less common breeds. However it is important to stress that these studies were based on relatively small samples and were likely underpowered to identify all true breed associations regardless of this approach. Exploration of individual breed-disorder predispositions was not a primary objective of this study but rather to establish evidence for breed associations in general, in support of disorders that may have an inherited element. It remains interesting to note that the majority of breed-disorder over-representations seen were consistent with previously reported breed-disorder associations, including otitis externa in Cocker spaniels [67, 68], osteoarthritis in Labrador and Golden retrievers [69, 70] and dermatitis in Bulldogs, Boxers, Pugs and German shepherd dogs [47, 57]. As such the findings of the present study appear to support existing evidence for established breed-disorder associations and could direct future predisposition investigations using larger sample sizes [71].

### Limitations

This study aimed to develop and apply a standardised methodology for disorder assessment to maximise comparability of prevalence, duration and severity across disorders, but individual disorder assessments and comparisons remain sensitive to certain ‘critical control points’. As previously discussed, these include selection criteria for disorders to be assessed, specificity of case definitions, inclusion criteria, subjective interpretation of available clinical free text, availability of all required data for parameter estimation, criteria for assigning severity metric sub-scores and methodology for annual duration estimation. Nonetheless, the primary comparisons aimed to establish relative measures and scores rather than absolute numerical indices of health-related welfare. Defining SEHB level 2 in at least one breed as sufficient evidence to consider a disorder ‘breed-related’ did not constitute strong evidence for genetic inheritance/hereditability. However, in this study the interest was in comparing disorders with a tendency for association with certain breeds for various, not necessarily solely genetic, reasons. For example, environmental, epigenetic and socioeconomic factors may also be relevant if certain breeds tend to be owned by less experienced or affluent dog owners, those whose lifestyle/housing offers fewer opportunities for exercise, or are inclined to feed their pets in particular ways. Hence, it was not critical to seek the higher levels of evidence for genetic inheritance which remain relatively scarce within the available literature on canine genetics [14, 18, 19].

Broad diagnostic specificities applied within some disorder groupings may fall short of the fine-grained level achievable in referral or smaller-scale prospective studies. Some case definitions reflected common approaches in primary care practice, rather than confirmed diagnoses, e.g. lipoma diagnosis without pathological supporting evidence or osteoarthritis diagnosis without radiographic imaging. However, these wider case definitions better reflect the typically varied level of diagnostic certainty available to clinicians in the primary care setting and allowed researchers to harness the unique benefits of analytical scale offered by the VetCompass database.

Using data available within VetCompass in 2013 introduced certain potential limitations to the generalisability of study findings to all UK primary care practices. The study included a mix of private and corporate but with a predominance of corporate practices. This may have skewed the results towards the standards and policies of clinical management and care that are typical of these corporate practices. Additionally, the study represents the demographic breed structure in dogs in 2013. This structure of the UK veterinary dog population may change over time with consequent changes in the frequency and nature of the most common disorders. The age of the dataset could also mean that certain topical/relevant breed-disorder associations change, e.g. brachycephalic breeds are becoming increasingly popular in the UK. These breed types have strong, well-documented disorder associations that would therefore be expected to elevate the frequency of these disorders in the overall dog population over time. Future studies analysing more recent VetCompass datasets can provide valuable scope to reflect and compare current caseloads within both private and corporate veterinary settings.

All welfare metric scoring in this study was designed for disorder (rather than individual) level assessment, thus could not be used to reflect ranges in severity between individual cases of the same disorder. The authors recognise that the range of individual case severity within-disorder is an important area of research, and could provide opportunities to examine relative severity of a disorder between defined sub-groups of affected dogs (e.g. individual breeds). Appropriate re-framing of the proposed severity scoring system, or modification of published severity scoring systems such as the GISID [14] for practical application to VetCompass EHR data at individual dog level would expand the scope of future VetCompass studies to describe welfare impact at both individual, group and overall disorder levels. It should also be acknowledged that the current work focused on health related welfare impacts only and did not attempt to evaluate other elements of the animals’ welfare. Further, though the study identified seven specific metrics for assessment, it is acknowledged that not all of these might apply equally to all conditions. As such, the aim of the study was to offer transparent and consistent severity assessments. By providing the individual metric scores in addition to their combination, there remains the scope for those interpreting these scores to focus on one or more individual metrics, rather than all measures as deemed appropriate.

Generating an overall welfare impact score by summing selected indicators of prevalence, severity and duration may seem to contradict the published concept of population welfare impact as the product of these three key concepts [35, 36]. Taking a summative approach was considered a mathematically valid alternative, and allowed the inclusion of a zero score within the severity scoring system (to maintain distinction between none and few cases contributing data of relevance within individual severity metrics). While differential weighting of individual contributor factors was avoided here, a summative approach to welfare impact scoring still offers scope for mathematical weighting should future users wish to emphasise the importance of particular contributing factors on welfare impact score.

On balance, this large-scale epidemiological analysis of primary-care EHRs provides valuable evidence, complementary to that from more detailed, smaller scale studies undertaken prospectively or within referral/specialist caseloads. Specific controversies over disorder definitions or researcher judgements should not detract from the applicability of the disorder assessment approach across a range of conditions. Transparency of case definitions, data availability and criteria for individual metric calculation are key to the valid interpretation of findings from future applications of these methods.

## Practical implications

The practical implications of this study are many and varied. While not aimed directly at pet owners, findings reported can indirectly raise owner awareness through veterinary healthcare professionals, providing the latter with evidence to support care recommendations in priority areas of welfare concern which are routinely underestimated or dismissed by owners as unimportant. We also present a transferable framework for evidence-based disorder prioritisation, potentially useful for canine health and welfare stakeholders with funding to allocate to particular areas of canine health-related welfare concern. Applied to new (or expanded) lists of potential priority disorders, this tool could help canine charities, organisations or research institutions to target available resources at disorders with greatest population-level welfare impact. Strategic funding of public awareness campaigns, further research or lobbying for improvements to regulations governing the key areas of concern highlighted could ultimately raise standards of welfare in pet dogs across the UK. Results from VetCompass studies on breed-related disorders are directly contributing to the ongoing development of breed health strategies such as those of the Kennel Club’s Breed Health and Conservation Plans project [72] and the strategic framework aims of the Brachycephalic Working Group [73]. In addition, our research group are actively updating the accepted welfare constructs of animal welfare scientists through conferences such as UFAW [74].

## Conclusions

Eight of the most common breed-related conditions seen in dogs attending primary care veterinary practices were identified for assessment of disorder-specific health-related welfare impact. Novel assessment tools were developed and applied to evaluate EHR information from affected dogs to score and compare health-related welfare impact at a population level. Dental disorders, osteoarthritis and overweight/obese had highest overall VetCompass Welfare Impact scores. Severity metric sub-scores appear to support mismatches between veterinary and owner perceptions of obesity/excessive bodyweight and dental disease as significant canine health issues [51], suggesting benefits from improved owner awareness in these areas.

This study provides proof-of-concept for the applicability of standardised methods for population-level, health-related welfare impact assessment across a spectrum of canine conditions using EHR data. The VetCompass Programme offers substantial practical opportunities for evidence-based disorder prioritisation according to sound epidemiological principles [35, 36]. The proposed novel welfare impact assessment offers an evidence-based alternative to existing, expert opinion-based scoring methods, reflecting relative welfare burdens at the population-level based on real data. Disorder comparison via transparent consideration of all contributory metrics leaves scope for subsequent users of the results to place differential emphasis on individual contributory factors as desired. The proposed strategy is directly applicable to a broad range of disorder prioritisation scenarios, offering an evidence-based but flexible decision-making framework for stakeholders keen to maximise welfare improvement returns from investment of available resources. The VetCompass Welfare Impact assessment tools developed here could also be incorporated into prospective studies, providing unique or complementary perspectives on welfare effect within and between canine disorders.

## Methods

### Ethical approval

The project received ethical approval from the RVC Ethics and Welfare committee (RVC URN 2015 1369).

### Source of data

The VetCompass Programme collates de-identified EHR data from UK primary-care veterinary practices for epidemiological research. Collaborating practices can record summary diagnosis terms during episodes of care from an embedded VeNom code list [75]. Data fields held within the VetCompass database include species, breed, date of birth, sex, neuter status, insurance status, bodyweight, clinical information from free-text clinical notes, any summary diagnosis terms including VeNom codes recorded, and treatments/interventions sold in association with individual animals, all with associated recording dates.

### Selection of disorders for welfare impact assessment

Disorders were considered for assessment based on fulfilment of two criteria:

1. Relatively high disorder frequency in UK dogs. Disorders with highest estimated prevalence in UK veterinary-attending pet dogs were identified by review of previously published VetCompass analyses [46].
2. Acceptable evidence in the literature to suggest a disorder may show breed-relatedness, i.e. particular association with at least one canine breed, rather than broadly affecting all breeds/types within the canine population at a similar frequency. The authors determined that an acceptable level of evidence to define a disorder as breed-associated was attainment of a minimum level 2 on the Strength of Evidence for Hereditary Basis (SEHB) scale for at least one canine breed/type. SEHB level 2 requires ‘prior evidence of an apparent breed predisposition based on numerical over-representation/risk compared to a suitable baseline or reference population’ upon review of existing literature of relevance to that disorder [18].

Within the time restraints of this project it was possible to complete welfare impact assessment studies in eight of the most prevalent disorders meeting both these criteria.

### Study population

The study population included all dogs under veterinary care at VetCompass participating clinics between January 1st and December 31st 2013 (inclusive). Dogs with a) at least one EHR recorded during 2013 or b) at least one EHR recorded both before *and* after 2013 were defined as ‘under veterinary care during 2013’. An animal was considered to have an EHR if a VeNom diagnosis term, free-text clinical note, treatment or bodyweight was recorded.

### Disorder assessment strategy

Assessment of the welfare impact of each selected disorder on the UK dog population was undertaken via disorder-specific studies of standardised design, using VetCompass EHR data from 250 randomly selected cases recorded during 2013. Power calculations to report the prevalence of individual conditions with a precision of +/− 2–3% indicated each disorder study required approximately 200–250 cases per condition (95% confidence level, OpenEpi, Version 3).

A multi-stage approach was used for case selection. First, dogs in the study population likely to be affected by the disorder during 2013 were identified as ‘candidate cases’ (CCs), using keyword searches designed to achieve good sensitivity and specificity for case capture. Keyword searches were based on disorder-associated terms (full or partial words or groups of words) likely to be recorded in clinical notes, recorded as VeNom terms or within parameter fields of relevance to individual disorders (e.g. body condition score). Initial keyword selection was based around disorder-specific literature reviews, relevant VeNom diagnostic terms and expert opinion (obtained through consultation with specialist veterinary practitioners in a field relevant to each disorder). Search terms were combined into keyword search strategies which were then trialed, refined and expanded iteratively using additional terms suggested by natural language processing (NLP) searches [76]. NLP searches were based on words commonly occurring in the notes of sample cases originally identified by certain key disorder terms. At each stage, combinations of search terms were evaluated via review of EHR data from a pilot sample of the potential cases captured until a final set of search terms was established. Total CCs identified by the final search strategy and a list of unique identifiers were recorded.

Next, EHR data from a random sample of all CCs identified was manually reviewed against specified case definition criteria, to determine a disorder-specific study groups of 250 verified cases. A sub-group of all CCs identified by the final keyword search strategy was randomly sampled for potential inclusion. Randomisation was achieved using the RAND function in Microsoft Excel (2013) to assign each CC a random identification (ID) number. The CCs were then sorted by descending magnitude of ID. Maintaining this order, EHR data from individual CCs were manually reviewed in detail against the case definition to verify or reject disorder-affected status in 2013. For consistency, manual review was undertaken by an individual researcher, experienced in reviewing primary care veterinary clinical notes, according to protocols developed in consultation with other VetCompass research team members over a preliminary study design period. Case definitions for the selected disorders were finalised with reference to published literature. The main elements consistent across all disorder case definitions were as follows:

- EHR data must have provided a ‘clear, recorded diagnosis’ of the relevant disorder; i.e. the diagnosis must have been recorded in at least one clinical note from the year 2013 using accepted terms or via recognisable synonyms, abbreviations, misspellings or typing errors.
- Where relevant health parameters were embedded within the clinical records, values acceptable as directly indicative of disorder were stated (e.g. scores of 5/10 or above in a Body Condition Score field were accepted as indicating clinically overweight).
- Notable additional inclusion criteria were specified; e.g. dogs where 2013 notes indicated a permanent disorder-associated abnormality originating before 01.01.2013, resulted in the animal being classified as disorder positive during 2013 according to the relevant case definition.
- Examples of acceptable disorder-specific recording terminology and common conventions in note-taking were specified (e.g. ‘AGs++’, indicated that anal sacs were excessively full, thus abnormal).

Manual reviewing continued down the list until 250 cases were verified for inclusion in each disorder group. The case verification rate within the CC sample was calculated based on the number of CCs reviewed to achieve 250 cases (*250/total CCs reviewed to verify 250 cases*).

Data relevant to assessment of welfare impact and study group demography were extracted from the EHRs of all individual cases using both automated and manual extraction methods. Automated demographic data extraction identified breed (categorised by individual UK Kennel Club (KC) recognised breed or type, or as generic ‘Crossbreed’) and dates of birth and death. All additional clinical data required for welfare metric generation were extracted via structured manual case review using a bespoke software application (VetCompass App; see acknowledgements). Relevant data were extracted from cases using standardised study questions relating to severity and duration during EHR review (see measures described below), then data for each disorder group were pooled for generation of disorder-level welfare metrics, as described below. Case-finding and data interrogation were limited to data from 2013 in order to derive annual prevalence estimates and evaluate disorder severity and duration based on a consistent time period, for optimal comparability.

### Health related welfare metric generation

#### Prevalence

Annual period prevalences (i.e. the proportion of the population affected by each disorder at any point during the study year) were estimated via a scaling method, as previously described [77]. The proportion of cases verified from detailed review of EHR data from the randomly selected subset of CCs was used as a scaling factor to estimate the expected number of confirmed cases if all candidate cases had been reviewed. The prevalence was estimated as the expected number of cases if all candidate cases had been reviewed divided by the study VetCompass denominator. Ninety five per cent confidence intervals for annual period prevalence were calculated by standard approaches taking into account the sampling approach [78].

#### Disorder duration

A number of metrics were defined to reflect different aspects of disorder duration across the spectrum of diseases. Duration metrics included: median individual episode duration (days), median number of episodes per year, median total annual duration (median % of year affected), median age at first disorder presentation or diagnosis in 2013 and the proportion of each disorder group affected by the disorder for the full year (%).Metrics were generated as appropriate for individual disorders using clinical knowledge of their nature and patterns of occurrence where recorded in the EHR. Disorders with evidence of at least one start and end date available in clinical records, within the study period, were considered ‘episodic’. Disorders considered to be permanent or continuous, even with therapeutic management/intervention were considered ‘non-episodic’.

Duration metric calculation was approached separately for episodic and non-episodic conditions. For episodic conditions, the median episode duration (episodic disorders) was estimated using durations of all episodes of a disorder recorded in the EHRs in 2013. A case could contribute more than one episode to this calculation, or none if diagnosis and resolution dates were not available for any episodes. The median number of episodes per case was calculated directly from the number of episodes seen in cases in 2013. The median percentage of the year affected was calculated from the median number of episodes multiplied by the median episode duration during 2013 (as below). The range for duration was calculated from the minimum and maximum number of episodes and their respective durations*.* Where the upper limit was calculated as greater than 365 days this was reported as 100%. The interquartile range was not calculated for episodic disorders.

Median % of year affected (episodic disorders) = [(median number of episodes per case*median episode duration)/365]*100.

Range of % study year affected = (minimum number of episodes per case during year*minimum episode duration) to (maximum episodes per case during year*maximum episode duration).

For non-episodic conditions the median percentage of 2013 affected, was calculated directly from the affected periods of individual cases. For cases with a start date prior to 1st January 2013 and no evidence of death during the year this was classified as a 100% year duration. For dogs first diagnosed in 2013 the duration was from the start date until the end of 2013 or the date of death if there was evidence the animal died during 2013.

Additional duration metrics calculated (where relevant to individual disorders) included the proportion of cases with > 1 episodes per study year (episodic disorders only), the proportion of cases affected by the disorder for the entire year and the age at first disorder presentation or diagnosis in 2013.

#### Disorder severity

Seven individual severity metrics were defined based on both their potential to objectively reflect aspects of veterinarian and/or owner perceived disorder severity and the types of clinical data commonly recorded in EHRs (Table 5). The severity measures included the apparent imperative to present dogs with this disorder for veterinary care (i.e. primary vs partial presenting complaint vs incidental finding), the frequency of disorder-related primary care visits, disorder-related analgesia and anti-inflammatory therapy, the range of other (i.e. non-analgesic/anti-inflammatory) medical therapy used, the extent of disorder-related surgical procedures performed, presence of overnight primary-care hospitalisations and evidence of disorder-related referral.

**Table 5:** Disorder-level score allocation criteria for the seven individual severity metrics contributing to overall severity score. Sub-scores assigned at disorder-level, based on analysis of group data obtained via structured review of electronic health records from 250 affected dogs per disorder during 2013

Disorder-level sub-score category	*Severity metric 1:* Disorder as reason presented for veterinary care during year	*Severity metric 2:* Frequency of disorder-related primary care clinic visits during year	*Severity metric 3:* Chronicity of disorder-related analgesia (including AI) therapy during year	*Severity metric 4:* Range of other (non-analgesic/AI) therapeutic groups used for disorder treatment/management in year	*Severity metric 5:* Surgical depth of any disorder-related procedures performed under GA/sedation during year	*Severity metric 6:* Disorder-related overnight primary-care hospitalisations during year	*Severity metric 7:* Disorder-related referral during year
*Aims to reflect:*	*Owner perception of need/urgency of need for veterinary care*	*Veterinary opinion/owner perceived importance for repeated veterinary visits*	*Vet/owner perceived need for any pain relief and for how long (*i.e. *over shorter or longer periods of time)*	*Clinical indications and perception of extent of veterinary intervention required to manage the disorder*	*The level/depth of surgical intervention required to manage the disorder appropriately*	*Vet/owner perceived requirement for veterinary in-patient level care related to the disorder*	*Vet/owner perceived requirement for referral-level care related to the disorder*
*Assigned based on assessment of group data showing:*	*Evidence for the disorder as a primary or secondary/partial presenting complaint, or an incidental finding*	*Median number of disorder-related EHR visit entries per case in the year*	*Proportion of cases prescribed disorder-related analgesic drug therapy, for up to 14 days or longer*	*Proportion of cases prescribed/sold no, 1–2, or > 2 different non-analgesic/non-AI therapeutic groups related to the disorder*	*Proportion of cases within different maximum disorder-related surgical intervention categories*	*Median number of disorder-related overnight primary-care hospitalisations per case during the year*	*Proportion of cases with evidence for at least one disorder-related referral during the year.*
Sub-score 0	N/A (At least one disorder-related visit during year required for selection as case)	N/A (At least one disorder-related visit during year required for selection as case)	0 cases prescribed disorder-related analgesia/AIs during year	0 cases were sold disorder-related, non-analgesic/AI therapeutic interventions	*No procedures:* 0 cases underwent disorder-related procedures under GA or sedation	0 cases with disorder-related overnight hospitalisation at a primary care clinic	0 cases referred for investigation/management/treatment of the disorder
Sub-score 1	Disorder only ever noted as an incidental finding in > 50% of cases	Median 1–2 disorder-related visits per case	< 50% cases prescribed some form of analgesia / AI therapy for the disorder	Under 50% (but > 0%) of cases were sold ANY disorder-related, non-analgesic/AI therapeutic interventions	*Few procedures:* Under 50% (but > 0%) of cases underwent disorder-related procedures under GA or sedation	At least 1 case with an overnight hospitalisation but median overnight disorder-related hospitalisations per case: 0	Up to 1% of study cases referred for investigation/management/treatment of the disorder
Sub-score 2	> 50% of cases presented with disorder as a partial presenting complaint, at most	Median 3–5 disorder-related visits per case	50% or more cases prescribed 1–14 consecutive days of analgesia / AI therapy for the disorder	> 50% of cases were sold disorder-related, non-analgesic/AI interventions from 1 or 2 distinct therapeutic groups	*Many minor procedures:* 50% or more cases underwent procedures no deeper than skin/CT/MM under GA or sedation	Median disorder-related overnight hospitalisations per case during study year: 1 to 5	1–3% of study cases referred for investigation/management/treatment of the disorder
Sub-score 3	> 50% of cases presented at least once with disorder as primary complaint	Median > 5 disorder-related visits per case	> 50% of cases prescribed > 14 consecutive days analgesia / AI therapy for the disorder	> 50% of cases were sold disorder-related, non-analgesic/AI interventions from > 2 distinct therapeutic groups	*Many major procedures:* > 50% cases underwent procedures deeper than skin/CT/MM under GA or sedation	Median disorder-related overnight hospitalisations per case: > 5	> 3% of study cases referred for investigation/management/treatment of the disorder
If no single relevant results category contains > 50% of dogs……	If % of dogs in categories 1 and 2 (above) combined exceeds % in categories 2 and 3 combined: Assign sub-score 2 If % of dogs in categories 2 and 3 combined exceeds % in categories 1 and 2 combined: Assign sub-score 3	N/A	If % of dogs in categories 1 and 2 (above) combined exceeds % in categories 2 and 3 combined: Assign sub-score 2 If % of dogs in categories 2 and 3 combined exceeds % in categories 1 and 2 combined: Assign sub-score 3	N/A	If % of dogs in categories 1 and 2 (above) combined exceeds % in categories 2 and 3 combined: Assign sub-score 2 If % of dogs in categories 2 and 3 combined exceeds % in categories 1 and 2 combined: Assign sub-score 3	N/A	N/A

Abbreviations: *AI* Anti-inflammatory, *GA* General anaesthesia, *CT* Connective tissue, *MM* Mucous membrane, *N/A* Not applicable

Data relevant to each metric were manually extracted from the EHRs of all cases using a structured set of questions, as previously described. Disorder-level sub-scores on a four-point ordinal scale (0–3, indicating increasing severity level) were assigned at a disorder (not individual animal) level. These were based on the combined findings within each disorder, to reflect an ‘average’ severity level for the disorder. A zero score was assigned only where no cases contributed to a given severity metric (e.g. no cases referred or hospitalised overnight during 2013). Individual metrics and sub-score criteria are summarised in Table 5. For each disorder, sub-scores were reported individually and summed to generate an overall disorder level severity score (maximum 21). This composite scoring approach was adapted from concepts used in the previously published GISID severity scoring system [14], modified for scoring at a disorder (rather than individual) level.

#### VetCompass welfare impact scoring

Of the welfare metrics explored annual period prevalence (%), median proportion of year affected (%) and disorder overall severity score (maximum 21) were selected as the representative measures of disorder prevalence, duration and severity, respectively. Disorder-level scores for these three metrics were mathematically standardised and used to generate a novel health-related VetCompass Welfare Impact (VWI) score, directly comparable between disorders. For each disorder, absolute metric values scores were transformed to index values (expressed on a scale from 0 to 1) by setting the highest scoring disorder assessed as the denominator within each metric. For example the prevalence index value for a given disorder was the annual period prevalence for that disorder divided by the annual period prevalence for the disorder with highest prevalence. This allowed direct comparison of disorders within each key metric and ensured equal contribution of each metric to the disorder-level VWI score. Using the highest scoring disorder as the metric baseline provided a baseline relevant to the specific set of disorders being compared, rather than using external benchmarks of frequency, duration and severity which could distort this comparison. Disorder-specific prevalence, duration and severity indices were summed to generate summative VWI scores, reflecting the population-level welfare impact of each disorder relative to the others assessed in this study [79]. VWI scores were ranked and tabulated alongside the key contributing welfare metric values.

### Numerical breed-disorder associations

For each disorder, breed–specific annual period prevalence (breed-specific prevalence) estimates were also calculated for breeds with ≥5 cases using the breed-specific expected case numbers and total relevant breed counts in the VetCompass study population denominator. This provided key within-breed annual period prevalences alongside all-breed annual period prevalence for each disorder. Evidence for potential breed-disorder associations was generated by comparing breed-specific estimates to overall (all-breed) annual period prevalence within individual disorders. A breed-specific:all-breed annual period prevalence ratio > 1 without overlap of 95% confidence intervals was accepted as sufficient evidence for breed predisposition in accordance with the SEHB scale, Level 2 [18]. Exploration of individual breed-disorder predispositions was not a primary objective of this study. Rather the assessment technique above was designed to establish evidence for any apparent breed links (thus breed-associated status in general) and to further support existing evidence for an inherited element in certain disorders.

### Statistical analysis

Categorical data used to allocate scores for welfare metrics were summarised using numbers of cases (n) and proportion of study group (%), whilst quantitative data were assessed graphically for normality and summarised with median, interquartile range and range, as appropriate [78]. Confidence intervals for proportions reported were calculated by standard methods [78].

## Abbreviations

AI: Anti-inflammatory
BVA: British Veterinary Association
CC: Candidate case
CI: Confidence interval
CKCS: Cavalier King Charles Spaniel
CT: Connective tissue
DALY: Disability-Adjusted Life Year
EHR: Electronic Health Record
GA: General anaesthesia
GSD: German Shepherd dog
IQR: Interquartile range
JRT: Jack Russell terrier
KC: Kennel Club
KCS: King Charles Spaniel
MM: Mucous membrane
N/A: Not applicable
NLP: Natural Language Processing
PDSA: Peoples Dispensary for Sick Animals
SEHB: Strength of Evidence for Hereditary Basis
UK: United Kingdom
VeNom: Veterinary Nomenclature
VWI: VetCompass Welfare Impact
WALY: Welfare-Adjusted Life Year
WHWT: West Highland White Terrier
WI: Welfare Impact
